# Supplementary material for: Time-restricted feeding promotes muscle function through purine cycle and AMPK signaling in Drosophila obesity models
Source: Nat Commun. 2023 Feb 21;14:949. doi: 10.1038/s41467-023-36474-4 (PMC9944249; doi:10.1038/s41467-023-36474-4)
Supplement: Supplementary file 1 — Supplementary Information [file 41467_2023_36474_MOESM1_ESM.pdf]

Supplementary Materials for

Time-restricted feeding promotes muscle function through purine cycle and AMPK signaling in Drosophila obesity models

Melkani et al., Correspondence and requests for materials should be addressed to G.C.M

(Email: girishmelkani@uabmc.edu)

This PFD file includes:

Supplementary Figures 1 to 12

Figure S1

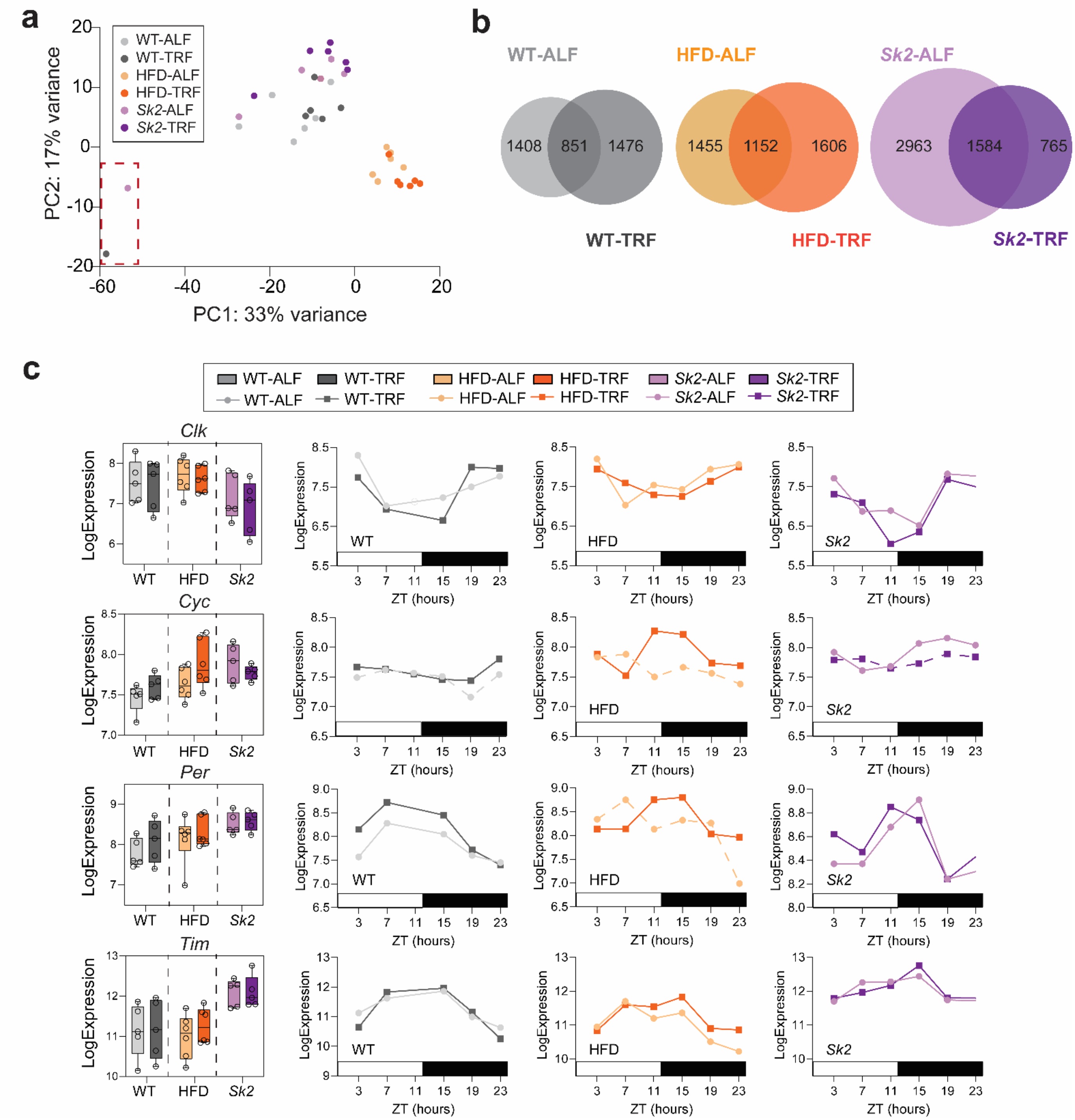

Figure S1: Overview of transcriptome data in WT, HFD, and *Sk2* models.
(a) PCA plot of skeletal muscle transcriptome data. The red rectangle indicated the two outliers (WT-TRF-ZT11 and *Sk2*-ALF-ZT23) and data from the two outliers were removed from all downstream analyses.
(b) Rhythmic skeletal muscle transcripts identified under ALF and TRF in WT, HFD, and *Sk2* flies.
(c) Expression level of *Clk*, *Cyc*, *Per,* and *Tim* under ALF and TRF in WT, HFD, and *Sk2* flies. Left panel, N = 5 time points in WT and *Sk2*. N = 6 time points in HFD. Right panel, expression levels at each time point. Empirical_JTK rhythmicity detection was presented as a solid line (Benjmanini-Hochberg adjusted *p*-value ≤ 0.05) or dash line (Benjmanini-Hochberg adjusted *p*-value > 0.05).

Figure S2

a

	Drosophila Ortholog	Human Ortholog	Fold Change (TRF / ALF) WT	HFD	*Sk2*	Annotated Function
Up Genes	*CG6188*	*Gnmt*	2.80	6.40	2.39	Glycine N-methyltransferase
	CG6385	*Sardh*	1.35	2.47	1.44	Sarcosine dehydrogenase
	CG5955	*Tdh*	1.60	2.02	1.64	Predicted to have L-threonine 3-dehydrogenase activity
	CG6806	N/A	3.25	3.46	2.23	Larval serum protein 2
	CG5896	N/A	1.31	1.68	1.37	Gram-positive specific serine protease
Down Genes	*CG7997*	*Gla*	-1.63	-1.73	-1.48	Predicted to have alpha-galactosidase activity
	CG1942	*Dgat2*	-1.51	-1.93	-1.55	Diacylglycerol O-acyltransferase 2
	CG13992	N/A	-5.4	-5.33	-4.04	Unknown

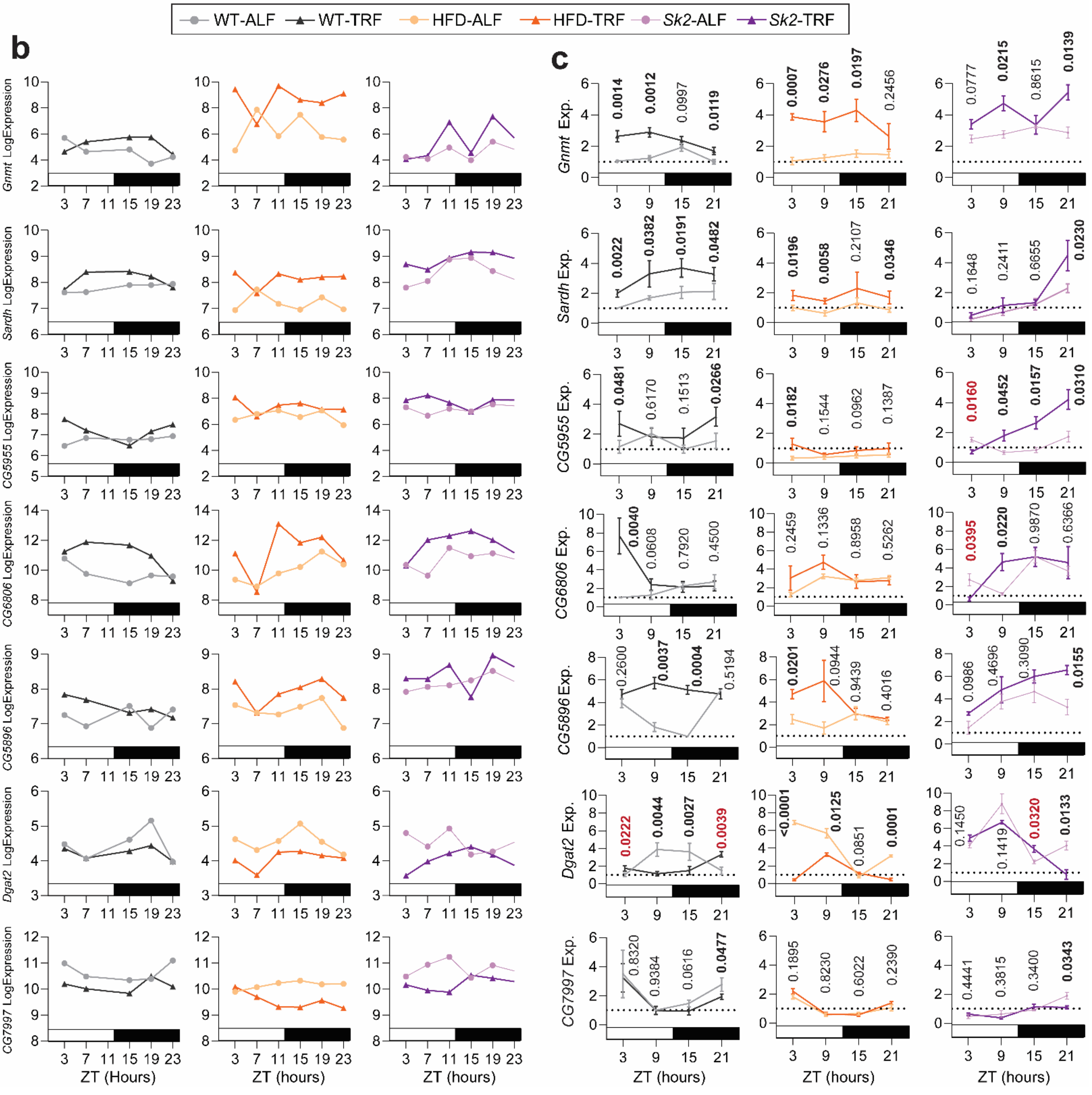

Figure S2: Profiles of the significantly upregulated genes across WT, HFD, and *Sk2* models.
(a) A table of differentially expressed genes shared across WT, HFD, and *Sk2* models.
(b) Temporal expression level of *Gnmt*, *Sardh*, *CG5955, CG6806, CG5896, CG7997, and Dgat2* under ALF and TRF in WT, HFD, and *Sk2* flies. Two outliers are removed.
(c) qPCR validation of temporal expression of *Gnmt*, *Sardh*, *CG5955, CG6806, CG5896, CG7997, and Dgat2* from IFMs under ALF and TRF in 3-week-old WT, HFD, and *Sk2* flies from independent experiments. The relative expression levels were normalized to the trough in WT-ALF. *CG13992* has low expression and cannot be detected robustly using qRT-PCR. N=3 biologically independent samples. Mean ± SEM. Two-sided unpaired t-test. Black *p*-value indicates that the significant expression change is aligned with transcriptomic data (Supplementary Fig. 2b), otherwise labeled as red.

Figure S3

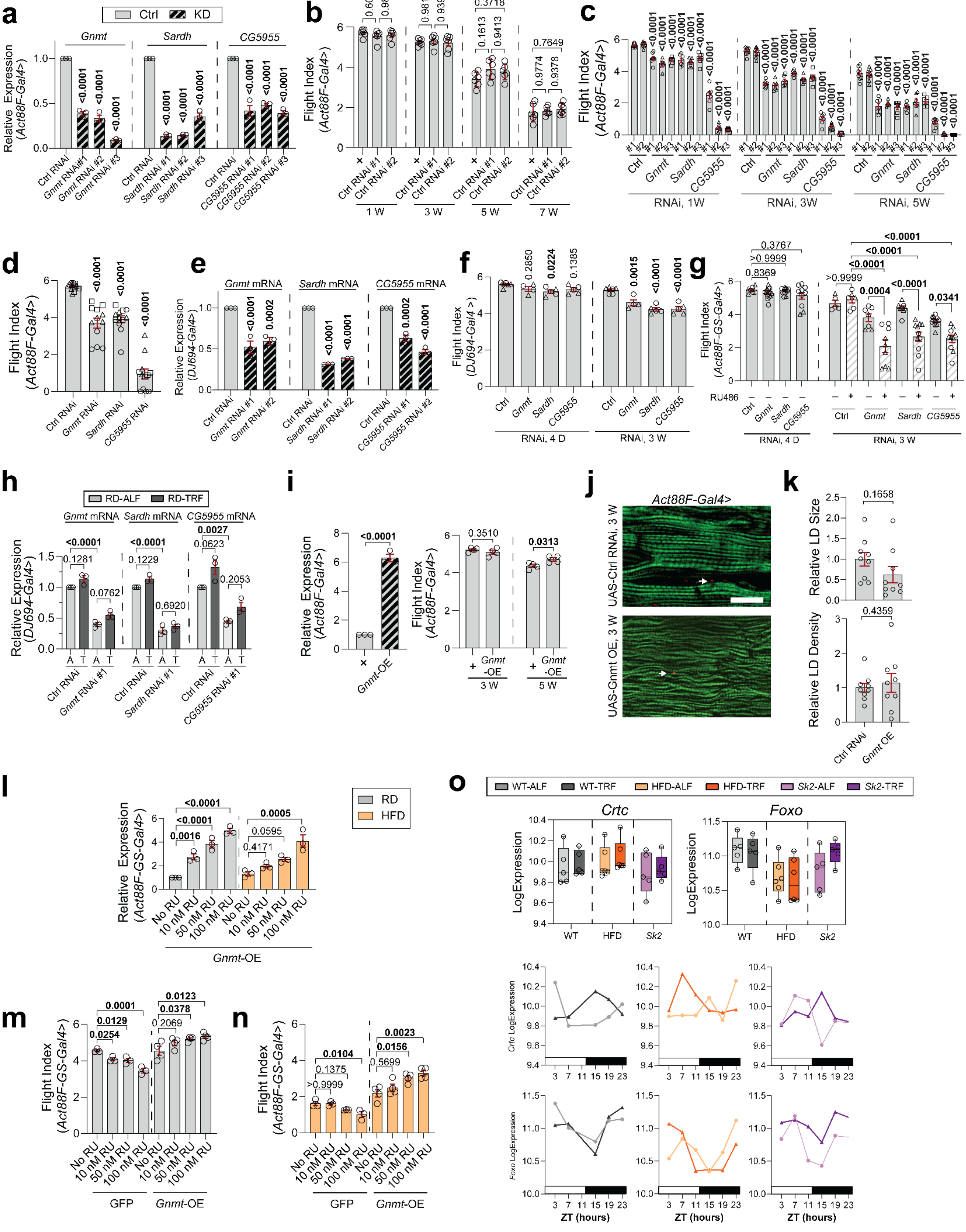
a
Ctrl
KD
Gnmt
Sardh
CG5955
Relative Expression (Act88F-Gal4>)
Ctrl RNAi
Gnmt RNAi#1
Gnmt RNAi #2
Gnmt RNAi #3
Sardh RNAi #1
Sardh RNAi #2
Sardh RNAi #3
CG5955 RNAi #1
CG5955 RNAi #2
CG5955 RNAi #3
b
Flight Index (Act88F-Gal4>)
Ctrl RNAi #1
Ctrl RNAi #2
1 W
3 W
5 W
7 W
c
RNAi, 1W
RNAi, 3W
RNAi, 5W
d
Ctrl RNAi
Gnmt RNAi
Sardh RNAi
CG5955 RNAi
e
Gnmt mRNA
Sardh mRNA
CG5955 mRNA
Relative Expression (DJ694-Gal4>)
f
Flight Index (DJ694-Gal4 >)
RNAi, 4 D
RNAi, 3 W
g
Flight Index (Act88F-GS-Gal4>)
RU486
h
RD-ALF
RD-TRF
i
Relative Expression (Act88F-Gal4>)
Gnmt-OE
j
Act88F-Gal4>
UAS-Ctrl RNAi, 3 W
UAS-Gnmt OE, 3 W
k
Relative LD Size
Relative LD Density
Ctrl RNAi
Gnmt OE
l
RD
HFD
Relative Expression (Act88F-GS-Gal4>)
No RU
10 nM RU
50 nM RU
100 nM RU
m
GFP
n
o
WT-ALF
WT-TRF
HFD-ALF
HFD-TRF
Sk2-ALF
Sk2-TRF
Crtc
Foxo
LogExpression
WT
HFD
Sk2
Crtc LogExpression
Foxo LogExpression
ZT (hours)

Figure S3: Profiles of common upregulated genes and evaluation of potential transcription factors.
(a) Relative expression of *Gnmt*, *Sarah,* and *CG5955* in the corresponding KD driven by *Act88F* in 3-week-old female flies. Three independent RNAi lines were used. Quantifications were done by qRT-PCR. Mean ± SEM is plotted for three technical replicates. Two independent experiments were performed with similar results, but one representative experiment is shown. One-way ANOVA with Sidak post hoc tests.
(b) Flight performance of *Act88F*/+, *Act88F*>Ctrl RNAi #1 and *Act88F*>Ctrl RNAi#2 from 1, 3, 5, and 7-week-old female flies. Flight performance was comparable among controls. Flight indices were indicated as cohorts of 10-20 flies. # of cohorts per RNAi line per age group = 6; # of flies per RNAi line per age group = 76-102. Details of precise N are in Source Data. Mean ±SEM. One-way ANOVA with Sidak post hoc tests.
(c) Flight performance of 1, 3, 5, and 7-week-old female flies with *Gnmt*, *Sardh,* and *CG5955* KD driven by *Act88F*. The data plotted as individual lines are identical to Fig. 2d. Flight Indices were plotted as cohorts of 10-20 flies. Results from independent RNAi lines were indicated with the symbol circle, triangle, or square. # of cohorts per RNAi line per age group = 7-9; # of flies per RNAi line per age group= 100-170. Details of precise N are in Source Data. Mean ± SEM. One-way ANOVA with Sidak post hoc tests.
(d) Flight performance of 1-week-old male flies with *Gnmt, Sardh,* or *CG5955* KD driven by *Act88F*. Three independent RNAi lines per gene were tested. Flight indices were indicated as cohorts of 10-20 flies. Results from independent RNAi lines were indicated with the symbol circle, triangle, or square. # of cohorts per RNAi line per condition = 4; # of flies per genotype = 107-184. Details of precise N are in Source Data; Mean ± SEM. One-way ANOVA with Sidak post hoc tests.
(e) Relative expression of *Gnmt, Sardh,* or *CG5955* in 3-week-old female flies with corresponding KD driven by *DJ694*. Two independent RNAi lines were used. Quantifications were done by qRT-PCR. Mean ± SEM is plotted for three technical replicates. Two independent experiments were performed with similar results, but one representative experiment is shown. One-way ANOVA with Sidak post hoc tests.
(f) Flight performance of 4-day- and 3-week-old male flies with *Gnmt*, *Sardh*, or *CG5955* KD driven by *DJ694*. Two independent RNAi lines per gene were tested. Flight indices were indicated as cohorts of 10-20 flies. Results from independent RNAi lines were indicated with the symbol circle or triangle. # of cohorts per RNAi line per condition = 2-3; # of flies per genotype per age group = 73-117. Details of precise N are in Source Data. Mean ± SEM. One-way ANOVA with Sidak post hoc tests.
(g) Flight performance of female flies with *Gnmt*, *Sardh*, or *CG5955* KD using *Act88F-GS* at 4 days and 3 weeks of age. RU486-induced (100nM) KD was started on day 4. Two independent RNAi lines per gene were tested each line is indicated by the symbol (circle and triangle). Flight indices were indicated as cohorts of 9-24 flies. # of cohorts per RNAi line per age group per condition= 2-4. # of flies per genotype per age group per condition = 86-171. Details of precise N are in Source Data. Mean ± SEM. Two-way ANOVA with Sidak post hoc tests.
(h) Relative expression of *Gnmt*, *Sardh*, and *CG5955* in 3-week-old female ALF and TRF flies with corresponding KD by *DJ694*. Quantifications were done by qRT-PCR. Mean ± SEM is plotted for three technical replicates. Two independent experiments were performed with similar results, but one representative experiment is shown. One-way ANOVA with Sidak post hoc tests.
(i) Relative expression of *Gnmt* (left panel) was tested in 3-week-old female flies and flight indices (right penel) of 3- and 5-week-old female flies with *Gnmt* overexpression driven by *Act88F*. Flight indices were indicated as cohorts of 10-20 flies. # of cohorts per genotype per age group = 4. # of flies per genotype per age group = 54-68. Details of precise N are in Source Data; Mean ±SEM. Two-sided unpaired t-tests.
(j) Fluorescence images of the IFMs from 3-week-old females with *Gnmt* overexpression driven by *Act88F*. Phalloidin (green) and Nile Red (red puncta) staining compared with age-matched control. Mean ± SEM. The scale bar is 20 µm.
(k) Intramuscular lipid quantification of 3-week-old females (lipid droplet size and density) showed a significant reduction in lipid droplet size upon *Act88F*-driven overexpression of *Gnmt* compared to age-matched control. The control plotted here is the same control in Fig. 2f and 2g. Mean ± SEM, N = 9 from three flies' IFM per genotype. Two-sided unpaired t-tests.
(l) Relative expression of *Gnmt* in 3-week-old female flies with *Gnmt* overexpression using *Act88F-GS* driver. Experimental flies were fed with RD or HFD supplemented with titrated concentrations of RU486 (0, 10, 50 100nM). Mean ± SEM is plotted for three technical replicates. Two independent experiments were performed with similar results, but one representative experiment is shown. One-way ANOVA with Sidak post hoc tests.
(m) Flight performance of *Act88F-GS*-driven *Gnmt* overexpression in 3-week-old female flies. Experimental flies were fed with RD supplemented with titrated concentrations of RU486 (0, 10, 50 100nM). Flight indices were indicated as cohorts of 10-20 flies. # of cohorts per condition = 3-4; # of flies per group = 30-73. Details of precise N are in Source Data. Mean ± SEM. One-way ANOVA with Sidak post hoc tests.

(n) Flight performance of *Act88F-GS*-driven *Gnmt* overexpression in 3-week-old female flies. Experimental flies were fed with HFD supplemented with titrated concentrations of RU486 (0, 10, 50 100nM). Flight indices were indicated as cohorts of 10-20 flies. # of cohorts per condition = 3-4; # of flies per group = 41-74. Details of precise N are in Source Data. Mean ± SEM. One-way ANOVA with Sidak post hoc tests.
(o) Expression level of gene *Crtc and Foxo* under ALF and TRF in WT and obese models. N = 5 time points in WT and *Sk2*. N = 6 time points in HFD. Source data are provided as a Source Data file.

Figure S4

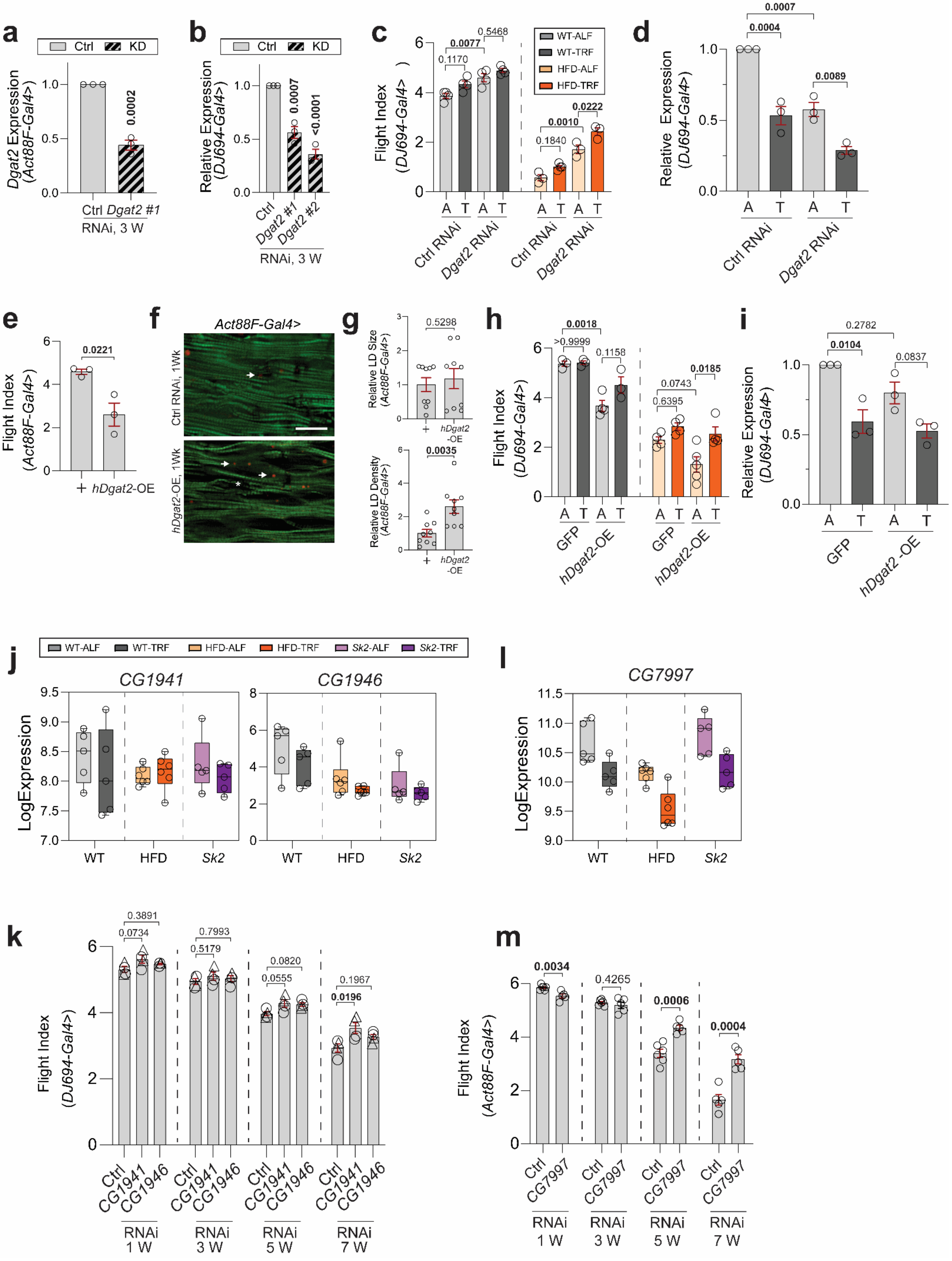

a
Ctrl
KD
Dgat2 Expression (Act88F-Gal4>)
0.0002
Ctrl Dgat2 #1
RNAi, 3 W
b
Relative Expression (DJ694-Gal4>)
0.0007
<0.0001
Ctrl
Dgat2 #1
Dgat2 #2
RNAi, 3 W
c
Flight Index (DJ694-Gal4>)
WT-ALF
WT-TRF
HFD-ALF
HFD-TRF
Ctrl RNAi
Dgat2 RNAi
d
Relative Expression (DJ694-Gal4>)
e
Flight Index (Act88F-Gal4>)
+ hDgat2-OE
f
Act88F-Gal4>
Ctrl RNAi, 1Wk
hDgat2-OE, 1Wk
g
Relative LD Size (Act88F-Gal4>)
Relative LD Density (Act88F-Gal4>)
h
Flight Index (DJ694-Gal4>)
GFP
hDgat2-OE
i
Relative Expression (DJ694-Gal4>)
GFP
hDgat2 -OE
j
WT-ALF
WT-TRF
HFD-ALF
HFD-TRF
Sk2-ALF
Sk2-TRF
CG1941
CG1946
LogExpression
WT
HFD
Sk2
l
CG7997
LogExpression
k
Flight Index (DJ694-Gal4>)
Ctrl
CG1941
CG1946
RNAi 1 W
RNAi 3 W
RNAi 5 W
RNAi 7 W
m
Flight Index (Act88F-Gal4>)
Ctrl
CG7997

Figure S4: Profiles of commonly downregulated genes across WT, HFD, and *Sk2* models.

(a) Relative expression of *Dgat2* in 3-week-old female flies with *Act88F*-driven *Dgat2* KD. Quantifications were done by qRT-PCR. Mean ± SEM is plotted for three technical replicates. Two independent experiments were performed with similar results, but one representative experiment is shown. Two-sided unpaired t-tests

(b) Relative expression of *Dgat2* in 3-week-old female flies with *DJ694*-driven *Dgat2* KD were quantified with qRT-PCR. Mean ± SEM is plotted for three technical replicates. Two independent experiments were performed with similar results, but one representative experiment is shown. One-way ANOVA with Sidak post hoc tests.

(c) Flight performance of 5-week-old female flies upon *DJ694*-driven *Dgat2* KD under ALF and TRF in RD or HFD. Flight indices were indicated as cohorts of 10-20 flies. # of cohorts per genotype per condition = 3-4. # of flies per genotype = 32-55. Details of precise N are in Source Data. Mean ±SEM. Two-way ANOVA with Sidak post hoc tests.

(d) Relative expression of *Dgat2* in 3-week-old female flies upon *DJ694*-driven *Dgat*2 KD under ALF and TRF. Mean ± SEM is plotted for three technical replicates. Two independent experiments were performed with similar results, but one representative experiment is shown. Two-way ANOVA with Sidak post hoc tests.

(e) Flight performance of 3-week-old female flies with h*Dgat2* overexpression using *Act88F* driver. Flight indices were indicated as cohorts of 10-20 flies. # of cohorts per genotype = 3. # of flies per genotype = 46-56. Details of precise N are in Source Data. Mean ±SEM. Two-sided unpaired t-tests.

(f) Fluorescence images of IFMs from 1-week-old females with *Act88F*-driven h*Dgat2* overexpression upon staining with phalloidin (green) and Nile Red (red puncta) compared with age-matched control. The scale bar is 20 µm.

(g) Intramuscular lipid quantification of 1-week-old females (lipid size and density) showed a significant reduction in lipid droplet size upon *Act88F*-driven h*Dgat2* overexpression compared to age-matched control. N = 9 from three flies' IFM per genotype. Mean ±SEM. Two-sided unpaired t-tests.

(h) Flight performance of 3-week-old female flies upon *DJ694*-driven *hDgat2* OE under ALF and TRF in RD or HFD. Flight indices were indicated as cohorts of 10-20 flies. # of cohorts per genotype per condition = 3-5. # of flies per genotype = 39-77. Details of precise N are in Source Data. Mean ±SEM. Two-way ANOVA with Sidak post hoc tests.

(i) Relative expression of *Dgat2* in 3-week-old female flies upon *DJ694*-driven *hDgat*2 OE under ALF and TRF. Mean ± SEM is plotted for three biologically independent replicates. Two independent experiments were performed with similar results, but one representative experiment is shown. Two-way ANOVA with Sidak post hoc tests.

(j) Expression level of *Dgat2* predicted paralogs *CG1941* and *CG1946* under ALF and TRF in WT and obese models. N = 5 time points in WT and Sk2. N = 6 time points in HFD.

(k) Flight performance of 1-, 3-, 5-, and 7-week-old female flies with *DJ694*-driven KD of *Dgat2* predicted paralogs *CG1941 and CG1946.* Two independent RNAi lines per gene were tested. Flight indices were indicated as cohorts of 10-20 flies. Results from independent RNAi lines were indicated with the symbol circle or triangle. # of cohorts per RNAi line per age group = 3; # of flies per genotype per age =63-80. Details of precise N are in Source Data. Mean ±SEM. One-way ANOVA with Sidak post hoc tests.

(l) Expression level of *CG7997* under ALF and TRF in WT and obese models. N = 5 time points in WT and Sk2. N = 6 time points in HFD.

(m) Flight performance of 1-, 3-, 5-, and 7-week-old female flies with *DJ694*-driven *CG7997* KD. One RNAi line was used. Flight indices were indicated as cohorts of 10-20 flies. # of cohorts per genotype per age group = 5-6; # of flies per genotype per age = 69-102. Details of precise N are in Source Data. Mean ±SEM. Two-sided unpaired t-tests. Source data are provided as a Source Data file.

Figure S5

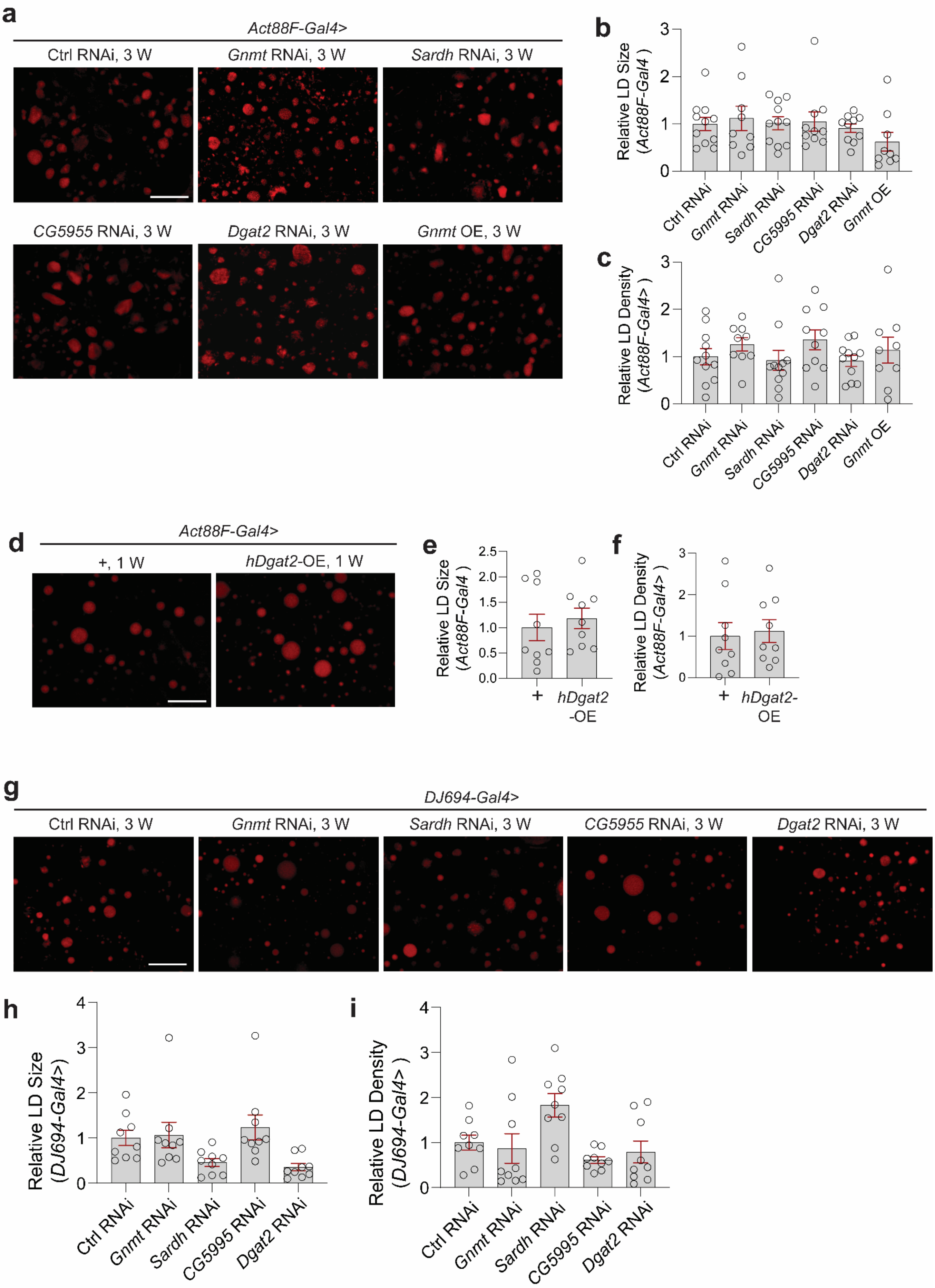

a
Act88F-Gal4>
Ctrl RNAi, 3 W
Gnmt RNAi, 3 W
Sardh RNAi, 3 W
CG5955 RNAi, 3 W
Dgat2 RNAi, 3 W
Gnmt OE, 3 W
b
Relative LD Size (Act88F-Gal4)
Ctrl RNAi
Gnmt RNAi
Sardh RNAi
CG5995 RNAi
Dgat2 RNAi
Gnmt OE
c
Relative LD Density (Act88F-Gal4>)
d
Act88F-Gal4>
+, 1 W
hDgat2-OE, 1 W
e
Relative LD Size (Act88F-Gal4)
+
hDgat2 -OE
f
Relative LD Density (Act88F-Gal4>)
+
hDgat2- OE
g
DJ694-Gal4>
Ctrl RNAi, 3 W
Gnmt RNAi, 3 W
Sardh RNAi, 3 W
CG5955 RNAi, 3 W
Dgat2 RNAi, 3 W
h
Relative LD Size (DJ694-Gal4>)
Ctrl RNAi
Gnmt RNAi
Sardh RNAi
CG5995 RNAi
Dgat2 RNAi
i
Relative LD Density (DJ694-Gal4>)

Figure S5: Fluorescence images of abdomen fat body of flies with knockdown or overexpression of common DEGs.

(a) Fluorescence images of the abdomen from 3-week-old females with *Act88F*-driven KD of *Gnmt*, *Sardh*, *CG5955,* and *Dgat2*, in addition to *Gnmt* overexpression upon probing with Nile Red (red puncta). The scale bar is 20 μm.

(b-c) Lipid quantification (lipid droplet size (b) and density (c)) showed no significant differences in lipid droplet size upon *Act88F*-driven KD of *Gnmt*, *Sardh*, *CG5955,* and *Dgat2* compared to age-matched control. In addition, no significant differences in lipid droplet size upon *Act88F*-driven *Gnmt* overexpression compared to age-matched control. N = 9 from three flies' IFM per genotype. Mean ± SEM. One-way ANOVA with Sidak post hoc tests.

(d) Fluorescence image of the abdomen from 1-week-old females with *Act88F*-driven overexpression of h*Dgat2*. The scale bar is 20 μm.

(e-f) Lipid quantification (lipid droplet size (e) and density (f)) showed no significant differences in lipid droplet size upon *Act88F*-driven h*Dgat2* overexpression compared to age-matched control. N = 9 from three flies' IFM per genotype. Mean ± SEM. Two-sided unpaired t-tests.

(g) Fluorescence images of the abdomen from 3-week-old females with *DJ694*-driven KD of *Gnmt*, *Sardh*, *CG5955,* and *Dgat2* upon probing with Nile Red (red puncta). The scale bar is 20 μm.

(h-i) Lipid quantification (lipid droplet size (h) and density (i)) showed no significant differences in lipid droplet size upon *DJ694*-driven KD of *Gnmt*, *Sardh*, *CG5955* and *Dgat2* compared to age-matched control. N = 9 from three flies' IFM per genotype. Mean ± SEM. One-way ANOVA with Sidak post hoc tests. Source data are provided as a Source Data file.

Figure S6

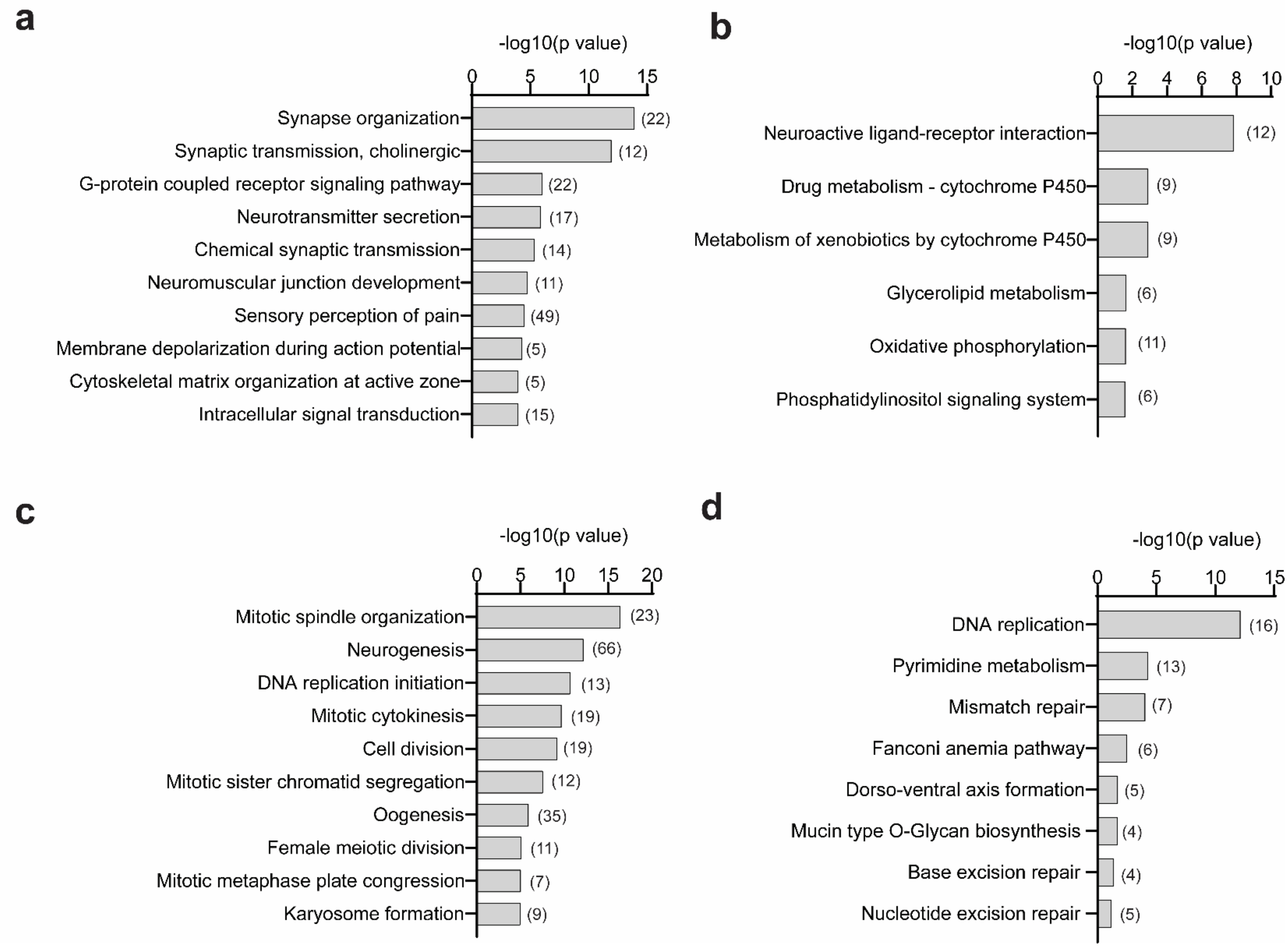

Figure S6: GO and Reactome analyses of significantly downregulated genes under TRF versus ALF in obesity models.

(a-b) GO analysis (a) and Reactome pathway analysis (b) of 636 genes that were significantly downregulated under TRF in the HFD model. Bar charts represent the - Log10 (*p*-value) of each enriched GO term and pathway. The number of genes identified in each GO term and pathway is shown in parentheses. No adjustments for multiple comparisons.

(c-d) GO analysis (c) and Reactome pathway analysis (d) of 579 genes that were significantly downregulated under TRF in the *Sk2* model. Bar charts represent the -Log10 (*p*-value) of each enriched GO term and pathway. The number of genes identified in each GO term and pathway is shown in parentheses. No adjustments for multiple comparisons.

Figure S7

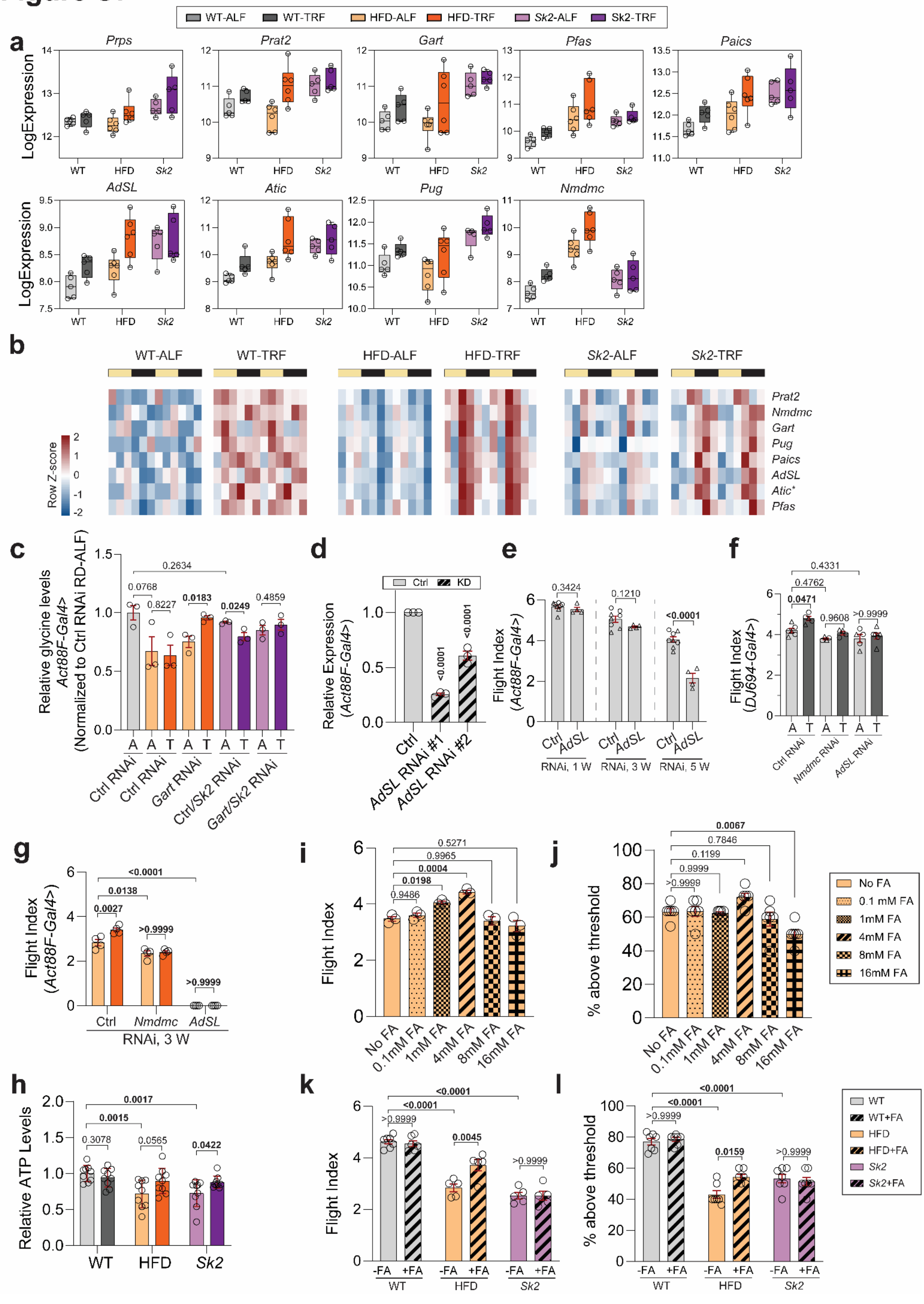

WT-ALF
WT-TRF
HFD-ALF
HFD-TRF
Sk2-ALF
Sk2-TRF
a
Prps
Prat2
Gart
Pfas
Paics
AdSL
Atic
Pug
Nmdmc
LogExpression
WT
HFD
Sk2
b
Row Z-score
Prat2
Nmdmc
Gart
Pug
Paics
AdSL
Atic*
Pfas
c
Relative glycine levels
Act88F-Gal4>
(Normalized to Ctrl RNAi RD-ALF)
0.2634
0.0768
0.8227
0.0183
0.0249
0.4859
Ctrl RNAi
Gart RNAi
Ctrl/Sk2 RNAi
Gart/Sk2 RNAi
d
Ctrl
KD
Relative Expression
(Act88F-Gal4>)
<0.0001
Ctrl
AdSL RNAi #1
AdSL RNAi #2
e
Flight Index
(Act88F-Gal4>)
0.3424
0.1210
<0.0001
RNAi, 1 W
RNAi, 3 W
RNAi, 5 W
f
Flight Index
(DJ694-Gal4>)
0.4331
0.4762
0.0471
0.9608
>0.9999
Ctrl RNAi
Nmdmc RNAi
AdSL RNAi
g
Flight Index
(Act88F-Gal4>)
<0.0001
0.0138
0.0027
>0.9999
Ctrl
Nmdmc
AdSL
RNAi, 3 W
h
Relative ATP Levels
0.0017
0.0015
0.3078
0.0565
0.0422
i
Flight Index
0.5271
0.9965
0.0004
0.0198
0.9486
No FA
0.1mM FA
1mM FA
4mM FA
8mM FA
16mM FA
j
% above threshold
0.0067
0.7846
0.1199
0.9999
>0.9999
No FA
0.1 mM FA
1mM FA
4mM FA
8mM FA
16mM FA
k
Flight Index
<0.0001
>0.9999
0.0045
-FA
+FA
l
% above threshold
0.0159
WT
WT+FA
HFD
HFD+FA
Sk2
Sk2+FA

Figure S7: Purine cycle genes under ALF and TRF in WT, HFD, and *Sk2* models.
(a) Expression levels of genes that are significantly upregulated under HFD-TRF versus HFD-ALF. N = 5 time points in WT and *Sk2*. N = 6 time points in HFD.
(b) Heatmap representation of rhythmic genes associated with purine cycle under HFD-TRF. Asterisks indicate genes that were unrhythmic under HFD-ALF but gained rhythmicity under HFD-TRF.
(c) Relative glycine levels of 3-week-old female flies with indicated genotypes under different diet regimens. N = three biologically independent replicates. Mean ± SEM. Two-sided unpaired t-tests.
(d) Relative expression of *AdSL* in 3-week-old female flies *Act88F*-driven *AdSL* KD with two independent RNAi lines were quantified with qRT-PCR. Mean ± SEM is plotted for three technical replicates. Two independent experiments were performed with similar results, but one representative experiment is shown. One-way ANOVA with Sidak post hoc tests.
(e) Flight performance of female flies with *AdSL* KD using *Act88F* and *UAS-AdSL* RNAi #2 at 1, 3, and 5 weeks of age. Data from two independent *AdSL* RNAi lines were plotted separately due to varying severity of phenotype and different KD efficiency. The control data here are the same as shown in Fig. 5e. Flight indices were indicated as cohorts of 10-20 flies. # of cohorts per genotype per age group = 4; # of flies per genotype per age = 51-143. Details of precise N are in Source Data. Mean ± SEM. Two-sided unpaired t-tests.
(f) Flight performance of 5-week-old female flies with *DJ694*-driven KD of *Nmdmc* and *AdSL* under RD-ALF and RD-TRF. Flight indices were indicated as cohorts of 10-20 flies. # of cohorts per genotype = 4-6; # of flies per genotype per condition = 54-84. Details of precise N are in Source Data. Mean ± SEM. Two-sided unpaired t-tests.
(g) Flight performance of 3-week-old female flies with *Act88F*-driven KD of *Nmdmc* and *AdSL* under HFD-ALF and HFD-TRF. N# = 30-50 females for each condition. Flight indices were indicated as cohorts of 10-20 flies. # of cohorts per genotype per age group = 4. # of flies per genotype per condition = 53-64. Details of precise N are in Source Data. Mean ± SEM. Two-sided unpaired t-tests.
(h) Relative ATP levels of 3-week-old WT, HFD, and *Sk2* flies under ALF and TRF. N = 9 biologically independent replicates. Mean ± SEM. Two-way ANOVA with Sidak post hoc tests.
(i) Flight performance of 10-day-old HFD-ALF female flies fed with food supplemented with 0.1 mM, 1 mM, 4 mM, 8 mM, and 16 mM folic acid (FA). Flight indices were indicated as cohorts of 10-20 flies. # of cohorts per condition= 3. # of flies per condition = 42-53. Details of precise N are in Source Data. Mean ± SEM. One-way ANOVA with Sidak post hoc tests.
(j) Climbing performance of 10-day-old HFD-ALF female flies fed with food supplemented with 0.1 mM, 1 mM, 4 mM, 8 mM, and 16 mM folic acid (FA). The climbing performance was indicated as cohorts of 10-13 flies. # of cohorts per condition = 6. # of flies per condition =60-65. Details of precise N are in Source Data. Mean ± SEM. One-way ANOVA with Sidak post hoc tests.
(k) Flight performance of 3-week-old WT, HFD, and *Sk2* female flies with or without folic acid supplement. Flight indices were indicated as cohorts of 10-20 flies. # of cohorts per condition= 5-7; # of flies per condition = 64-111. Details of precise N are in Source Data. Mean ± SEM. One-way ANOVA with Sidak post hoc tests.
(l) Climbing performance of 3-week-old WT, HFD, and *Sk2* female flies with or without folic acid supplement. Climbing performance was indicated as cohorts of 10-13 flies. # of cohorts per condition= 7. # of flies per condition = 70-81. Details of precise N are in Source Data. Mean ± SEM. One-way ANOVA with Sidak post hoc tests. Source data are provided as a Source Data file.

Figure S8

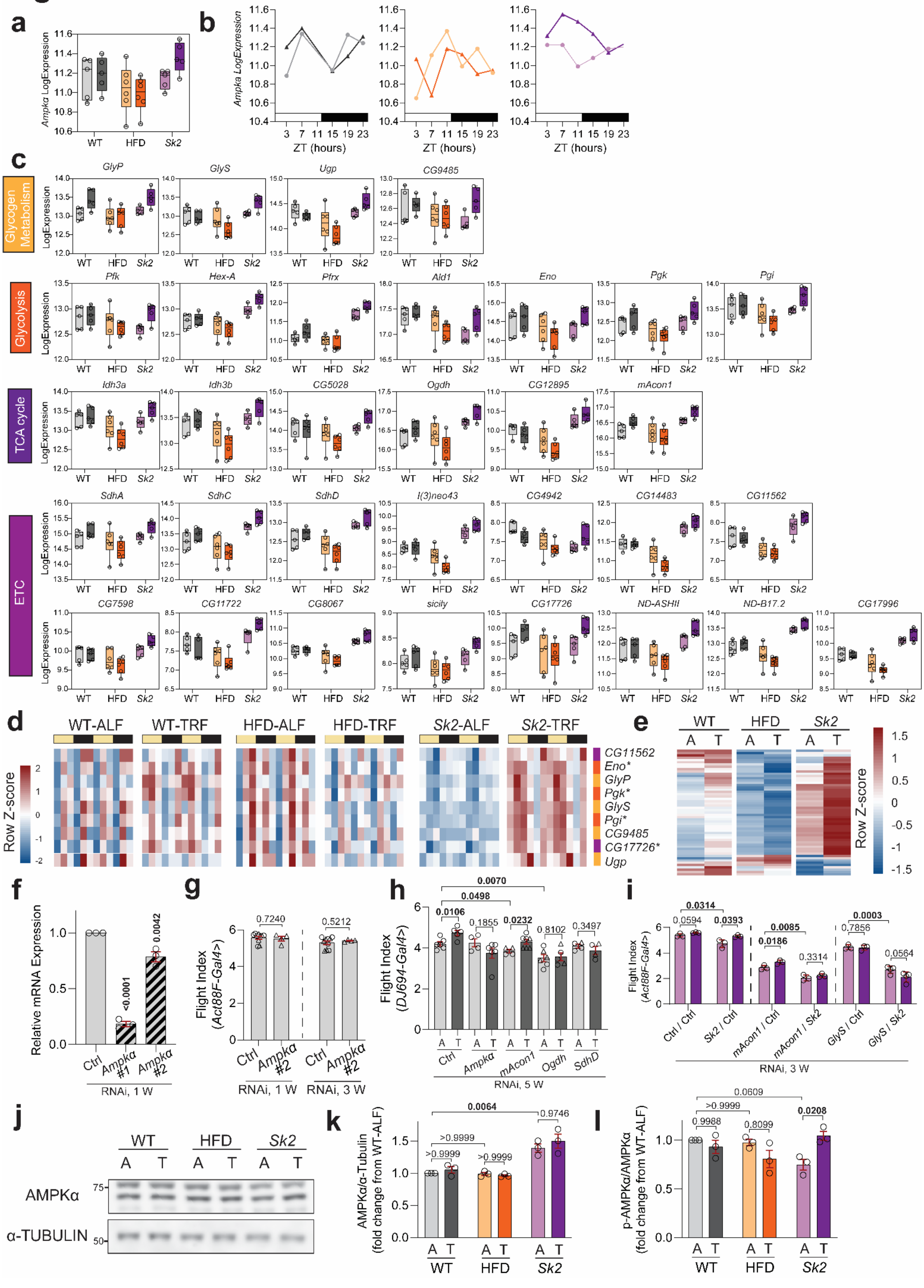
a
b
c
Glycogen Metabolism
Glycolysis
TCA cycle
ETC
GlyP
GlyS
Ugp
CG9485
Pfk
Hex-A
Pfrx
Ald1
Eno
Pgk
Pgi
Idh3a
Idh3b
CG5028
Ogdh
CG12895
mAcon1
SdhA
SdhC
SdhD
l(3)neo43
CG4942
CG14483
CG11562
CG7598
CG11722
CG8067
sicily
CG17726
ND-ASHII
ND-B17.2
CG17996
d
WT-ALF
WT-TRF
HFD-ALF
HFD-TRF
Sk2-ALF
Sk2-TRF
Row Z-score
e
f
Relative mRNA Expression
RNAi, 1 W
g
Flight Index (Act88F-Gal4>)
RNAi, 1 W
RNAi, 3 W
h
Flight Index (DJ694-Gal4>)
RNAi, 5 W
i
Flight Index (Act88F-Gal4>)
RNAi, 3 W
j
AMPKα
α-TUBULIN
k
AMPKα/α-Tubulin (fold change from WT-ALF)
l
p-AMPKα/AMPKα (fold change from WT-ALF)
WT
HFD
Sk2
ZT (hours)

Figure S8: TRF induces activation of AMPKα associated pathways in *Sk2* flies.

(a) Expression levels of *Ampkα* in WT, HFD, and *Sk2* flies under ALF and TRF. N = 5 time points in WT and *Sk2*. N = 6 time points in HFD.

(b) Expression level of *Ampkα* at each time point.

(c) Expression levels of genes from Fig. 5c in WT, HFD, and *Sk2* flies under ALF and TRF. N = 5 time points in WT and *Sk2*. N = 6 time points in HFD.

(d) Heatmap representation of the expression levels of rhythmic genes associated with AMPK signaling pathways under *Sk2*-TRF. Asterisks indicate genes that were unrhythmic under *Sk2*-ALF but gained rhythmicity under *Sk2*-TRF.

(e) Heatmap representation of the average expression levels of genes related to glycolysis, glycogen metabolism, TCA cycle, and, ETC with increased but not statistically significant under TRF versus ALF in the *Sk2* flies.

(f) Relative mRNA expressions of *Act88F*-driven *Ampkα* KD with two independent RNAi lines in 1-week-old female flies were quantified with qRT-PCR. Mean ± SEM is plotted for three technical replicates. Two independent experiments were performed with similar results, but one representative experiment is shown. One-way ANOVA with Sidak post hoc tests.

(g) Flight performance of *Ampkα* KD using *Act88F-Gal4* and *UAS-Ampkα* RNAi #2 at 1 and 3 weeks of age in female flies. Data from two independent *Ampkα* RNAi lines were plotted separately due to varying severity of phenotypes and different KD efficiency. The control data here are the same as shown in Fig. 6e. Flight indices were indicated as cohorts of 10-20 flies. # of cohorts per condition= 4-5; # of flies per genotype = 64-131. Details of precise N are in Source Data. Mean ± SEM. Two-sided unpaired t-tests.

(h) Flight performance of 5-week-old female flies with *DJ694*-driven KD of *Ampkα*, *mAcon1*, *Ogdh,* and *SdhD* under RD-ALF and RD-TRF. The control data is identical to Supplementary Fig. 7f. Flight indices were indicated as cohorts of 10-20 flies. # of cohorts per condition= 4-7; # of flies per genotype = 49-86. Details of precise N are in Source Data. Mean ± SEM. Two-sided unpaired t-tests.

(i) Flight index of 3-week-old flies with indicated genotypes (KD driven by *Act88F*) under ALF and TRF. Flight indices were indicated as cohorts of 10-20 flies. # of cohorts per condition= 3-4; # of flies per genotype = 34-54. Details of precise N are in Source Data. Mean ± SEM. Two-sided unpaired t-tests.

(j) Representative western blot of AMPKα levels (top) and α-TUBULIN (bottom), from 3-week-old female fly IFMs in WT, HFD, and *Sk2* flies under ALF (A) and TRF (T).

(k-l) Ratios of AMPKα/α-TUBULIN (j) and p-AMPKα/AMPKα(h) normalized to WT-ALF. N = 3 biologically independent replicates. Mean ± SEM. Two-way ANOVA with Sidak post hoc tests. Source data are provided as a Source Data file.

Figure S9

a

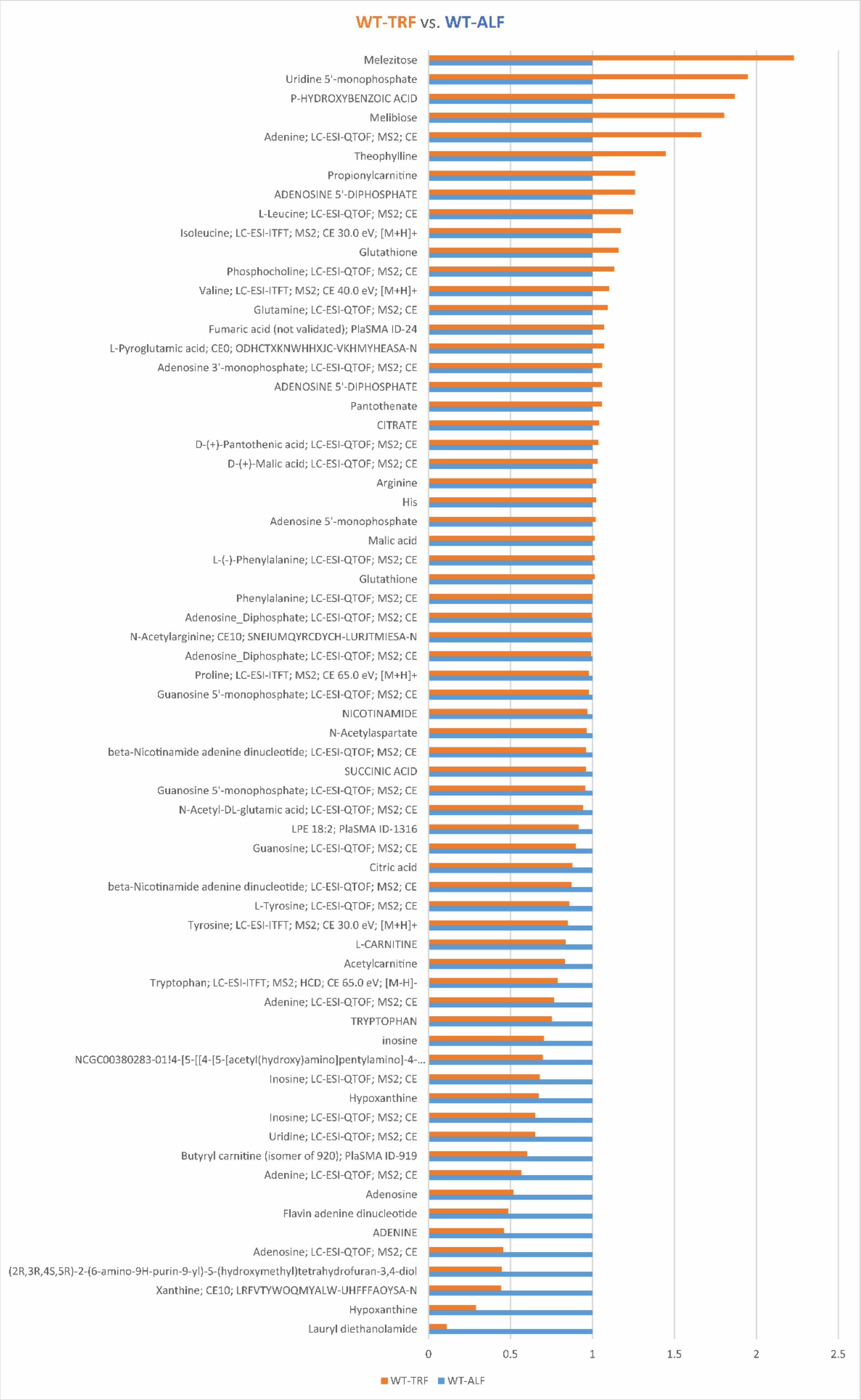

Figure S9: Metabolites in IFM (WT-TRF vs. WT-ALF).
(a) Untargeted metabolomics analysis was performed for WT-ALF (blue) and WT-TRF (orange) in 3-week-old female flies. The top 40 metabolites from the differential analysis were plotted with WT-ALF normalized to a value of 1-fold change.

Figure S10

a

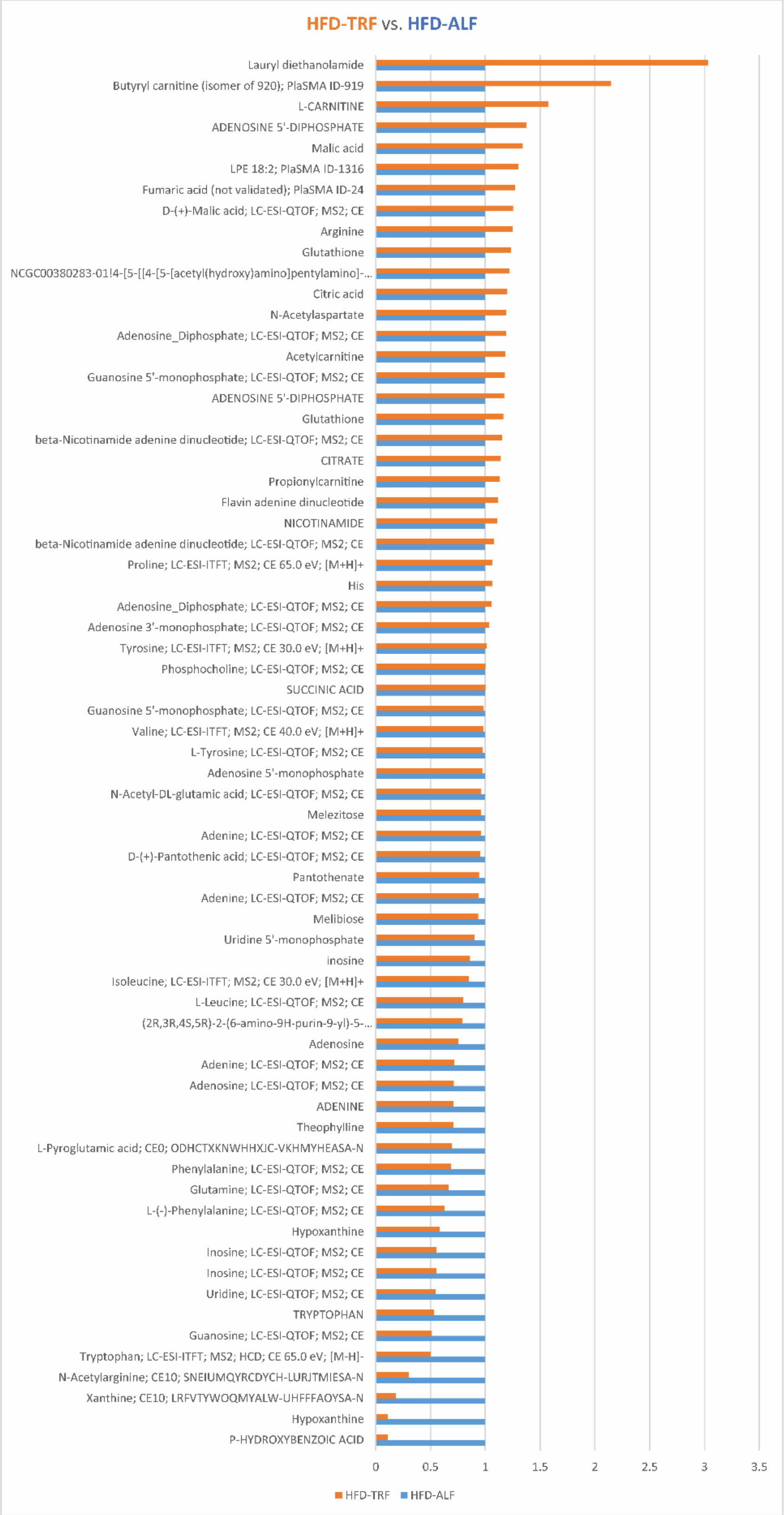

Figure S10: Metabolites in IFM (HFD-TRF vs. HFD-ALF).

(a) Untargeted metabolomics analysis was performed for HFD-ALF (blue) and HFD-TRF (orange) in 3-week-old female flies. The top 40 metabolites from the differential analysis were plotted with HFD-ALF normalized to a value of 1 fold change.

Figure S11

a

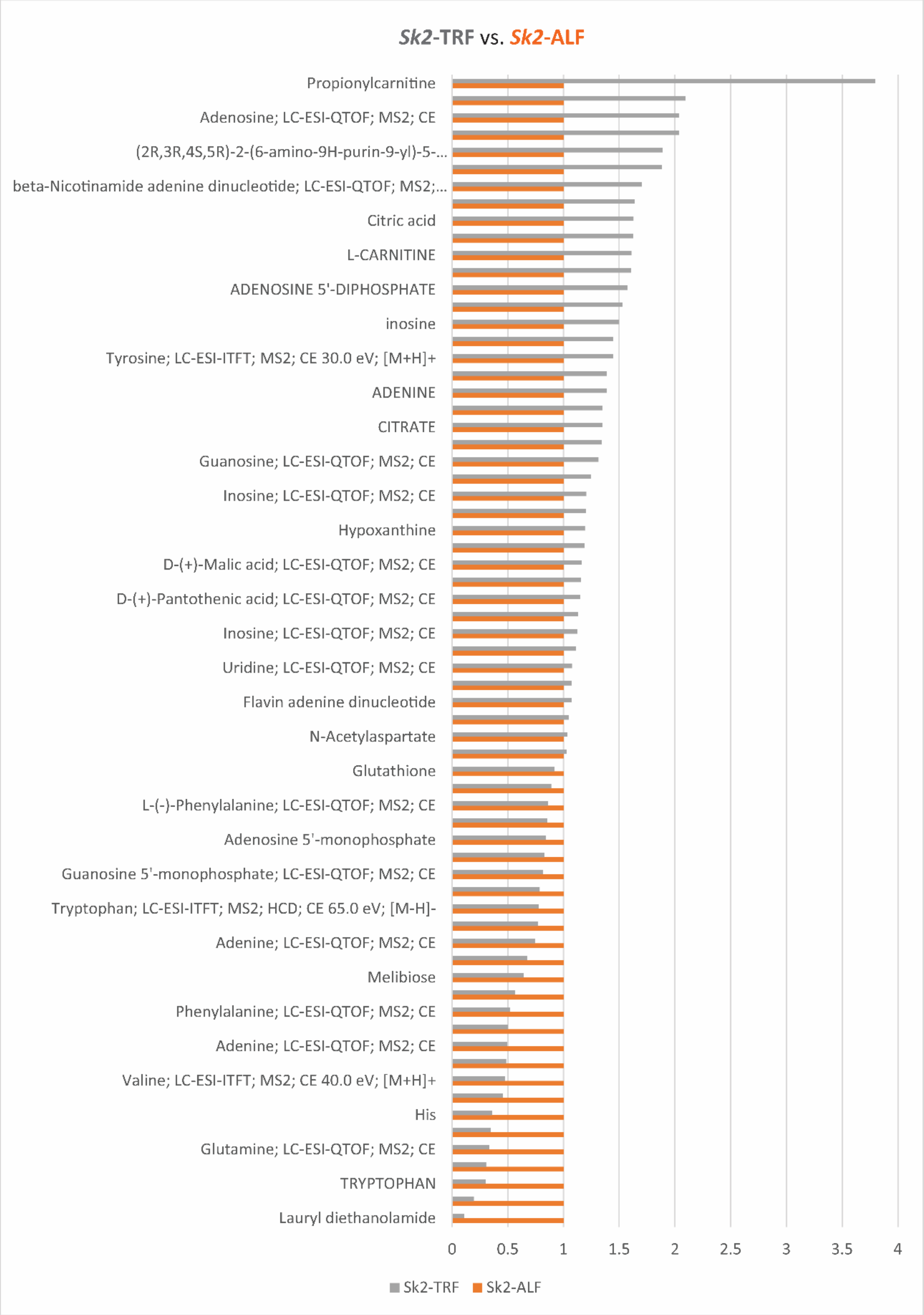

Figure S11: Metabolites in IFM (*Sk2*-TRF vs. *Sk2*-ALF).
(a) Untargeted metabolomics analysis was performed for *Sk2*-ALF (orange) and *Sk2*-TRF (gray) in 3-week-old female flies. The top 40 metabolites from the differential analysis were plotted with *Sk2*-ALF normalized to a value of 1 fold change.

Figure S12

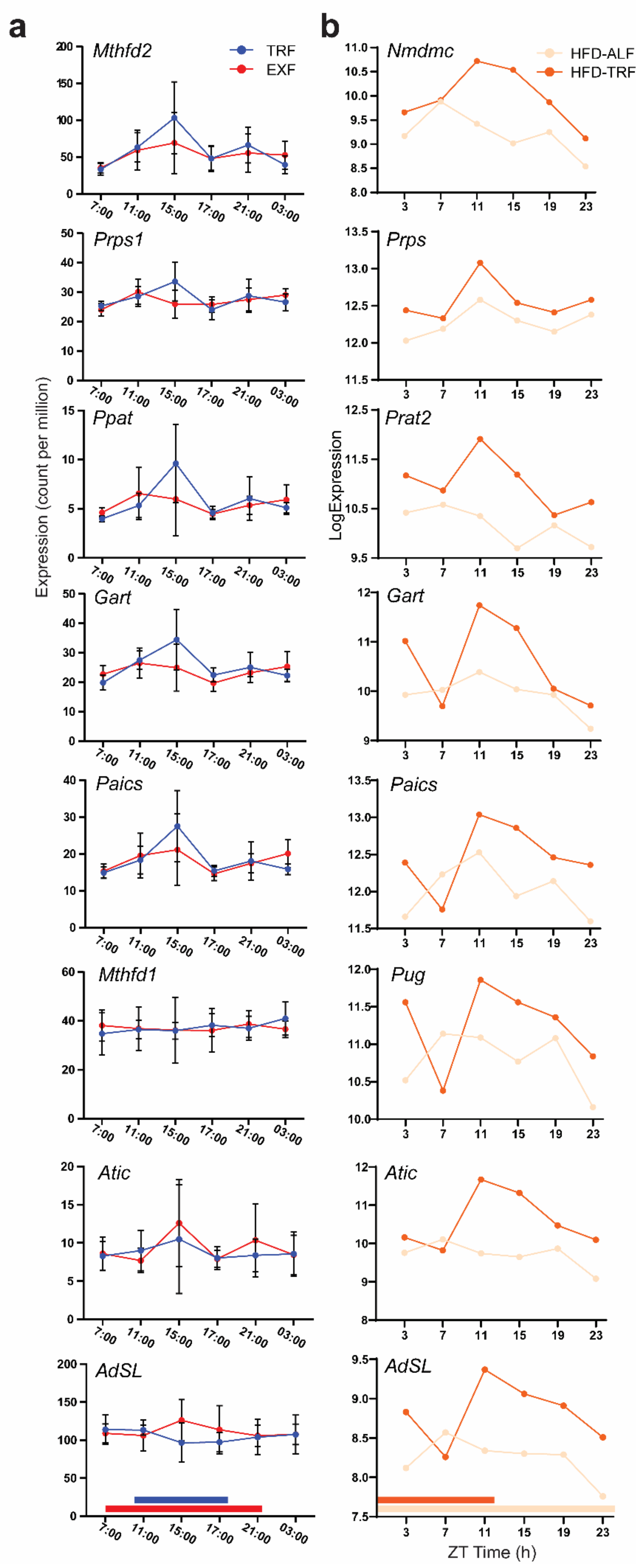

Figure S12: Comparison with skeletal muscle transcriptomic data from obese men under TRF.

(a) Expression levels of genes associated with purine cycle and folate cycle found in a human RNA-seq dataset under EXF and TRF[18]. Mean ± SEM is plotted for data collected from 6 men with overweight/obesity. 6 out of 11 participants are included as only these 6 participants have data collected for all time points from the original study. Participant IDs are 141, 312, 508, 536, 789, and 894.

(b) Expression levels of the same genes from (a) in our *Drosophila* RNA-seq dataset under HFD-ALF and HFD-TRF.
