# Supplementary material for: Time-restricted feeding promotes muscle function through purine cycle and AMPK signaling in Drosophila obesity models
Source: Nat Commun. 2023 Feb 21;14:949. doi: 10.1038/s41467-023-36474-4 (PMC9944249; doi:10.1038/s41467-023-36474-4)
Supplement: Supplementary file 3 — Description of Additional Supplementary Files [file 41467_2023_36474_MOESM3_ESM.pdf]

Description of Additional Supplementary Files

Supplementary Data 1: Expression (Log Norm Count) of Drosophila skeletal muscle transcripts under ALF and TRF in WT, HFD, and *Sk2* models.

Supplementary Data 2: Differentially expressed gene analysis from DeSeq2 under TRF versus ALF in WT.

Supplementary Data 3: Differentially expressed gene analysis from DeSeq2 under TRF versus ALF in HFD.

Supplementary Data 4: Differentially expressed gene analysis from DeSeq2 under TRF versus ALF in Sk2.

Supplementary Data 5: FPKM of genes upregulated in HFD-TRF.

Supplementary Data 6: FPKM of genes upregulated in Sk2-TRF.

Supplementary Data 7: Average FPKM of all genes in pathways seen in Human (restricted vs unrestricted) Genes.

Supplementary Data 8: Glossary of *Drosophila* orthologs of human genes and metabolite abbreviations.
